# Insights on Phytohormonal Crosstalk in Plant Response to Nitrogen Stress: A Focus on Plant Root Growth and Development

**DOI:** 10.3390/ijms24043631

**Published:** 2023-02-11

**Authors:** Nazir Ahmad, Zhengjie Jiang, Lijun Zhang, Iqbal Hussain, Xiping Yang

**Affiliations:** 1 State Key Laboratory of Conservation and Utilization of Subtropical Agro-Bioresources, Guangxi Key Laboratory of Sugarcane Biology, Guangxi University, Nanning 530004, China; 2 Department of Horticulture, Institute of Vegetable Science, College of Agriculture and Biotechnology, Zhejiang University, Hangzhou 310058, China; 3 National Demonstration Center for Experimental Plant Science Education, College of Agriculture, Guangxi University, Nanning 530004, China

**Keywords:** nitrogen stress, root growth, plant response, auxin, ethylene

## Abstract

Nitrogen (N) is a vital mineral component that can restrict the growth and development of plants if supplied inappropriately. In order to benefit their growth and development, plants have complex physiological and structural responses to changes in their nitrogen supply. As higher plants have multiple organs with varying functions and nutritional requirements, they coordinate their responses at the whole-plant level based on local and long-distance signaling pathways. It has been suggested that phytohormones are signaling substances in such pathways. The nitrogen signaling pathway is closely associated with phytohormones such as auxin (AUX), abscisic acid (ABA), cytokinins (CKs), ethylene (ETH), brassinosteroid (BR), strigolactones (SLs), jasmonic acid (JA), and salicylic acid (SA). Recent research has shed light on how nitrogen and phytohormones interact to modulate physiology and morphology. This review provides a summary of the research on how phytohormone signaling affects root system architecture (RSA) in response to nitrogen availability. Overall, this review contributes to identifying recent developments in the interaction between phytohormones and N, as well as serving as a foundation for further study.

## 1. Introduction

Enhancing crop yields and reducing environmental risks simultaneously is a massive challenge in sustainable agricultural development. In the past four decades, agricultural food production has doubled worldwide, and nitrogen (N) fertilizer use has increased seven-fold. Plants require N as a major macronutrient, and its availability has been recognized for years as a critical factor in crop production and food security [1,2]. The overuse of N fertilizers results in a 25–50% decrease in plant N uptake efficiency and a 60–70% decrease in plant nitrogen use efficiency (NUE) [3,4]. Furthermore, the overuse of N fertilizers not only negatively influences human health but also boosts agricultural production costs and entails environmental risks, such as water resource contamination and soil salinization [5].

Enhancing NUE in plants is crucial to enhancing yields and quality, reducing nutrient input costs, and improving soil, water, and air quality [6]. In addition to reducing fertilizer input costs, higher NUE by plants can reduce nutrient losses and increase crop production. The goal of improving crop NUE is to understand the whole system, from the macro level (agroecosystem) to the molecular level [7]. Thus, improving crop yields and reducing environmental risks requires a better understanding of how plants improve NUE.

N, an essential macronutrient for plants, is absorbed in two ways from the soil: inorganically, such as nitrate (NO_3_^−^) or ammonium (NH_4_^+^), or organically, mainly as free amino acids [8]. A major form of nitrogen in aerobic soils is nitrate, but nitrate availability can vary greatly over time and space depending on microbial activity and leaching [9,10]. In order to respond to the fluctuating NO_3_^−^ and NH_4_^+^ concentrations in the environment, plants have evolved numerous acquisition mechanisms for NO_3_^−^ and NH_4_^+^ with various affinities [11]. The amount of nitrogen absorbed from the soil and what is required for growth and development are balanced by plants through physiological and morphological responses. Moreover, root system architecture (RSA) changes can be modified for root adaptation to N availability. A variety of responses have been observed, including adjustments to the root growth [5], changes to the nitrogen uptake capacity [6,7], and changes in the root architecture [8,9]. Additionally, nutrient availability and hormone signals are coordinated to control RSA [12]. Hormones have been found to play an important role in root development as a reaction to NO_3_^−^ availability [13].

Considering the fact that higher plants contain multiple organs with distinct functions and nutritional requirements, coordination between these responses is necessary. As a result, communicating nutrient status between organs requires both local and long-distance signaling [14]. Several molecules have been implicated in this signaling process, including nitrate, amino acids, sugars, and phytohormones [15,16,17,18].

Phytohormones (PHs) are naturally occurring organic compounds that influence plant growth and development if found in small quantities [19,20]. Besides their basic functions in growth and development, light, temperature, salt, drought, pathogens, and nutrients are some of the environmental conditions associated with phytohormones [21,22,23,24]. In recent years, phytohormones have been revealed to play a critical role in plants’ ability to coordinate environmental signals with their internal growth and development processes [25,26,27,28]. It has been proposed that cytokinins (CKs), abscisic acid (ABA), auxin (AUX), ethylene (ETH), brassinosteroid (BR), strigolactones (SLs), jasmonic acid (JA), and salicylic acid (SA) act to coordinate the demand and acquisition of nitrogen [20,29,30,31]. Biotechnologists may use phytohormonal engineering as a powerful tool to enhance the nutritional value and economic sustainability of crops [26,32].

The modulation of nitrate uptake systems and the proliferation of lateral roots regulate nitrogen acquisition [33,34,35,36]. It is generally considered that transporters encoded by the *NRT1* and *NRT2* families have a low and high affinity for nitrate, respectively [37,38,39,40,41]. Numerous signals regulate the expression of *NRT* genes. For instance, a key component of the high-affinity nitrate transport system, *AtNRT2.1*, is stimulated by nitrate and sugars, while nitrogen assimilation products and CKs suppress it [42,43,44]. Different signals play important roles in the development of lateral roots, including nitrate, nitrogen assimilation products, ABA, AUX, ETH, CKs, BR, SLs, JA, and SA [45,46,47].

Our findings demonstrate the significance of nitrate as a signal that assists plants in responding to environmental changes by coordinating their life processes. This review aims to identify N-phytohormonal crosstalk networks and identify phytohormonal-regulated N uptake, transport, and absorption genes in plants that can be modulated by N availability. This study offers an overview of current findings in molecular mechanisms that interact with nitrate/ammonium and phytohormonal pathways to effectively govern plant growth and nutrition in Arabidopsis thaliana and other crop species.

## 2. Cytokinin (CK) Modulates RSA in Response to Nitrogen Stress

CKs are phytohormonal substances involved in nitrogen signaling and plant growth and development. According to an increasing body of evidence, macronutrients and cytokinins are complementary regulators of nutrient acquisition and distribution within the plant in response to its environment [48]. The discovery that nitrogen supply and CK levels are strongly related in *Hordeum vulgare* and *Urtica fissa* demonstrates a link between CK and nitrogen [49,50]. In *Plantago major*, exogenous CK treatment can partially resolve growth-limiting results caused by low nitrogen supply [51,52]. A similar association has been recorded in *Arabidopsis thaliana* (*A. thaliana*) [53].

The lateral root primordium (LRP) is initiated and organized by CKs, most likely by disrupting the auxin gradient and inhibiting its formation. CK has been shown to regulate the endocytic recycling of the auxin efflux carrier *PIN1* during lateral root (LR) development by redirecting it for vacuolar lytic degradation [54,55,56]. Aside from monitoring nitrate responses from root to shoot, CK acts as long-distance messengers. A significant amount of CK is produced when nitrate is applied, and as a result, that hormone may pass through the vascular bundles [57,58]. This leads to the accumulation of CK when nitrate increases the expression of CK biosynthesis genes *CYP735A* and *IPT3* [48,59]. As a result of the nitrate treatment, seven genes in the CK pathway are also activated [60,61,62].

**Figure 1:**
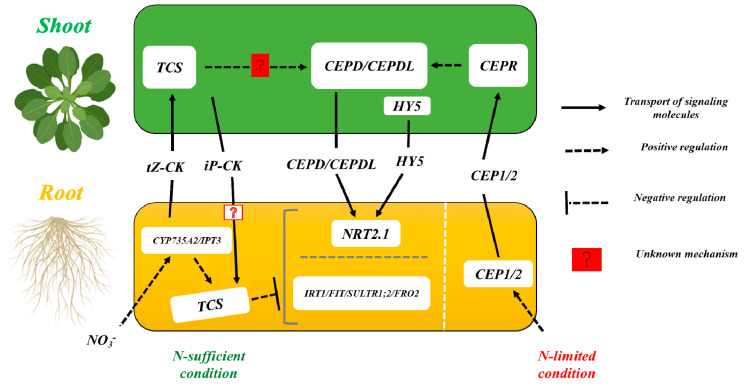
Cytokinin signaling pathways and other signaling players for nutrient uptake regulation. TCS—two-way component system for cytokinin signaling under nitrogen stress [66].

Nitrate feeding is proposed to increase the synthesis of *isopentenyl (iP)-type* CK in root phloem after *IPT3* is activated. *CYP735A* converts this type of CK into *trans-zeatin* (*tZ*), which facilitates leaf expansion and modulates gene expression changes in the shoot when exposed to high levels of nitrates (Figure 1). Aside from its proposed role as a long-distance signal, CK can also monitor local nitrogen (organ level) availability [63]. CK inhibits the accumulation of nitrate and ammonia transporters in nitrate-provided *Arabidopsis* plants’ roots. This mechanism may reflect a negative regulatory mechanism, which decreases nitrate consumption in non-limited conditions [64,65].

Recent studies have revealed that when roots are exposed to various nitrate sources, *C-TERMINALLY ENCODED PEPTIDE 1* (*CEP1*), *CEP RECEPTOR* (*CEPR*), and *CEP DOWNSTREAMS* (*CEPDs*) play key roles in regulating *NRT2.1* (Figure 1) [67]. *CEPDL2* can transport from the phloem to the cortex cells when *NRT2.1* is expressed after *CEP1/2* and *CEPDL2* have moved from shoot to root through the phloem [68].

In response to changes in the light environment, it has been documented that *ELONGATED HYPCOTYL 5* (*HY5*) translocates from the shoot to the root through the phloem and stimulates *NRT2.1* expression [69]. CKs may play a role in controlling global nutrient acquisition, while others (*CEPD*/*CEPDL* and *HY5*) may play more specialized roles in regulating nitrate uptake [70].

**Figure 2:**
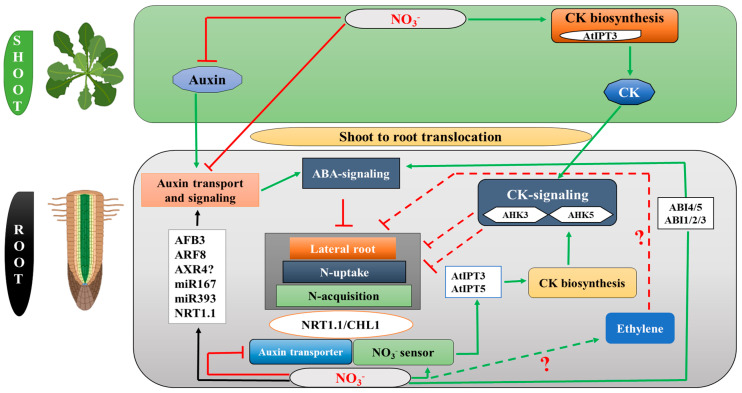
The interplay of nitrogen and phytohormones. The white box displays the identified molecular players. Positive, negative, and complex effects are indicated by green, red, and black lines, respectively. Known or putative links are marked with plain lines and dashed lines. Question marks indicate unconfirmed results.

A model was proposed for CK’s function as a root-to-shoot nitrate signal [71]. The *ARABIDOPSIS HIS KINASE* (*AHKs*, CK receptors), specifically (*AHK3* and *AHK5*), have been identified to play critical roles in root and shoot growth (Figure 2) [72]. Specific roles have been identified for each CK receptor. Notably, *AHK3* and *AHK5* play key roles in regulating the cell differentiation zone of the root meristem, root hair, and root elongation [73,74]. Table 1 summarizes a detailed summary of CK-related genes and their implications in RSA plasticity in response to various forms and concentrations of N.

## 3. Abscisic Acid (ABA) Modulates RSA in Response to Nitrogen Stress

Abscisic acid (ABA) is often referred to as a stress hormone but is actually a messenger that interprets both biotic and abiotic signals associated with the environment [81]. At the same time, it has been found that several plant species link ABA levels with nitrogen status. Although there is considerable evidence of a correlation between ABA levels and nitrogen status in several plant species, the relationship between both is not generally consistent [30,65,82,83,84,85]. For instance, there is no statistically significant difference in ABA levels of *A. thaliana* between high-nitrogen (HN) and low-nitrogen (LN) seedlings [86,87]. Whether changes in ABA content are important to nitrogen signaling is still uncertain, but it is becoming evident that ABA is involved in nitrogen signaling. Several studies indicate that ABA is involved in lateral root growth in response to a high nitrate supply in *A. thaliana* [88,89]. Mutants from *A. thaliana* with impaired ABA synthesis showed a decreased inhibition of lateral root formation after applying nitrate, suggesting that ABA signaling, at least partially, underpins the nitrate-induced root branching repression [90]. A typical plant response to ABA is the suppression of lateral root initiation [91].

Another group of *A. thaliana* mutants showing *ABA-insensitive lateral root initiation* (*LABI* mutants) has shown decreased sensitivity to nitrate resupply, supporting the idea of specific regulatory elements for ABA nitrate signaling [88]. Identifying *LABI* genes will be a milestone in understanding the mechanisms behind this inhibition effect.

**Figure 3:**
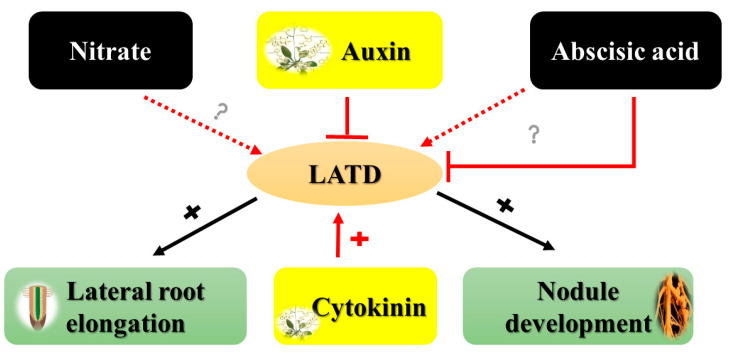
Review of *LATD* regulation, biochemical activity, and function. Black arrows indicate the involvement of *LATD* in nodule development and lateral root elongation. Red arrows and lines show up-regulation and down-regulation of *LATD* gene expression, respectively, by CK and AUX, and ABA. Dashed arrows indicate a proposed role of *LATD* (nitrate transport/ABA/another substance) (Adapted from [94]).

Furthermore, the rapid increase in ABA levels after nitrate treatment of barley roots may indicate that ABA directly controls the plant’s response to high nitrate levels. However, it may also be used to adapt to sudden changes in nitrate availability [92,93]. Further evidence for a correlation between ABA and nitrogen signaling was found in a recent study in a *Medicago truncatula lateral-root-organ-defective* (*LATD*) mutant [94]. The *LATD* mutant exhibits severe abnormalities in root meristem maintenance and development, which are rectified by exogenous ABA treatment [95,96]. Notably, the *LATD* mutant has a nitrate-insensitive primary root development, and the *LATD* gene encodes a transporter belonging to the *NRT1* (*PTR*) family [97,98]. Cytokinin stimulates, and auxin and ABA inhibit the expression of the *LATD* gene in root tips (Figure 3) [13].

In primary and lateral roots, *LATD* may regulate the activation of the meristems through a nitrate-ABA signaling pathway due to its homology to nitrate transporters [36]. This evidence also suggests a similar transition plays the same role in nodule formation. Given that *LATD* is expressed in both lateral roots and nodules, controlling *LATD* might be extremely important for maintaining a balance between lateral root and nodule development (Figure 3). *LATD* may perform a sensing function, possibly in response to nitrate or ABA, or it may be part of a sensing system. Future biological experiments should assist us in understanding how *LATD* works.

ABA also influences LR growth in the presence of localized NO_3_^−^ availability. It was documented that mutants lacking ABA biosynthesis (*aba1-1*, *2-3*, *2-4*, and *3-2*) as well as those lacking ABA insensitivity (*abi4-1*, *4-2*, and *5-1*) exhibited longer LR as a result of localized NO_3_^−^ supply than *wild type* (WT) (Figure 2) [99], suggesting that ABA acts as a negative regulator to modulate LR elongation. A summary of all the genes involved in N uptake and transport that are regulated by ABA is shown in Table 2.

## 4. Auxin (AUX) Modulates RSA in Response to Nitrogen Supply

Auxins are a group of important phytohormones that modulate plant development and morphology to environmental conditions and are vital under nitrogen stress [23,104,105]. Auxin accumulation is dependent on nitrogen sources [35,103]. Nitrogen can modulate auxin signals in both *A. thaliana* and rice [106,107]. Auxin signaling is upregulated in response to nitrogen starvation, as shown by the *DR5:: GUS* reporting method and reverse genetic approaches [20,49]. Auxin signaling is involved in primary and lateral root development in *Arabidopsis* in response to nitrate [108,109,110]. It has been widely thought that nitrogen signals are transmitted from shoot to root because auxin is transported basipetally and promotes lateral root growth [111,112]. As a result of high doses of nitrate being applied to maize, root growth is reduced, and less auxin is produced in the roots [113,114]. More important findings come from multiple transcriptome studies, indicating that the nitrate treatment impact genes are involved in auxin transport [115,116,117].

Similarly, a switch from a high-nitrate to a low-nitrate medium showed that auxin, in the roots, increased following lateral root growth in *A. thaliana*. Furthermore, plants grown in a medium with LN levels produced lower levels of auxins in their shoots than in a medium with HN [118,119]. Additionally, *Arabidopsis* seedlings cultivated under LN conditions produced greater root auxin levels than those grown under HN conditions, which indicates that dicot and monocot plants share a similar mechanism for controlling root auxin levels according to the nitrogen level of the plant [120]. However, it is critical to investigate this idea further because auxins do not reduce lateral root development suppression in plants growing in high-nitrate environments, implying that these hormones do not particularly control nitrate signaling. However, other signals may still be necessary to alter nitrate inhibitor effects [88].

A recent study in understanding auxin action suggests that the driving force of auxin-regulated growth and development is the concentration gradient and the differential sensitivity of various cell types, apart from the auxin levels in tissue. Cell-to-cell polar transport establishes the auxin gradient, and the differential sensitivity is achieved by modulating signaling components [121,122]. Recent research indicates that nitrogen signaling is regulated by the same or similar mechanisms [10,82,123,124]. It is now evident that auxins play a crucial role in regulating root system architecture at various stages, such as biosynthesis, signaling, transport, and auxin distribution.

Multiple studies have shown that auxin biosynthesis, transport, and accumulation are affected by different nitrogen regimes in maize [113], *Arabidopsis* [110], soybean [125], and rice [126,127]. Several important auxin-related regulatory modules that respond to nitrogen availability in *Arabidopsis* have been identified, including *TAR2*, a gene involved in auxin biosynthesis, auxin transporters such as *PIN1*, *PIN2*, *PIN4*, and *PIN7*, as well as molecular components that control their subcellular distribution [128]. As an N-responsive gene in the pericycle, *AUXIN RESPONSE FACTOR 8* (*ARF8*) was identified as involved in auxin signaling (Figure 2) [129]. The ratio between the initiation of LRs and their emergence is controlled by *ARF8* and its associated *microRNA167s* [130]. The nitrate transceptor, *NRT1.1*, is another mechanism that contributes to nitrogen–auxin interplay underpinning root system adaptation [131]. In NO_3_^−^-rich patches of soil, roots colonize, and this adaptation causes *AXR4*, a gene initially implicated in auxin signaling, to be activated (Figure 2) [132]. In addition, *AXR4* was found to target *AUX1* to the plasma membrane, indicating it plays an important role in auxin transport [133]. However, *AXR4*’s potential as a nitrate carrier or sensor remains to be seen. More recently, RSA has been controlled by a unique N-regulatory network based on *miR393/AFB3* [134].

It has been demonstrated that *NRT1.1*/*CHL1*, a nitrate sensor and dual-affinity nitrate transporter, contributes to the nitrate-induced expression of *AtIPT3* (Figure 2) [131]. Auxin transport by *NRT1.1* is thought to explain the discovery that a *chl1* mutant accumulates auxin in LRP [135], which suggests that nitrate and auxin signaling are interconnected. Table 3 summarizes all genes and their functions involved in AUX-regulated N uptake and transport.

## 5. Ethylene (ETH) Modulates RSA in Response to Nitrogen Stress

Ethylene is a master regulator of root growth and development due to its involvement in both *indole acetic acid* (*IAA*) trafficking and partitioning along the primary root and root growth [143]. Ethylene boosts *IAA* production by *triggering tryptophan aminotransferase* (TAA1) and *tryptophan aminotransferase-related* (*TAR1* and *TAR2*) enzymes, which have similar roles in ethylene responses [144]. It has been demonstrated that nitrogen consumption reduces leaf longevity primarily by altering the levels of ethylene in leaves. Perhaps a high N concentration increases the activity of enzymes involved in ethylene biosynthesis, accounting for the decrease in leaf N and leaf longevity caused by cobalt-chloride-induced reduction in ethylene biosynthesis [145].

**Figure 4:**
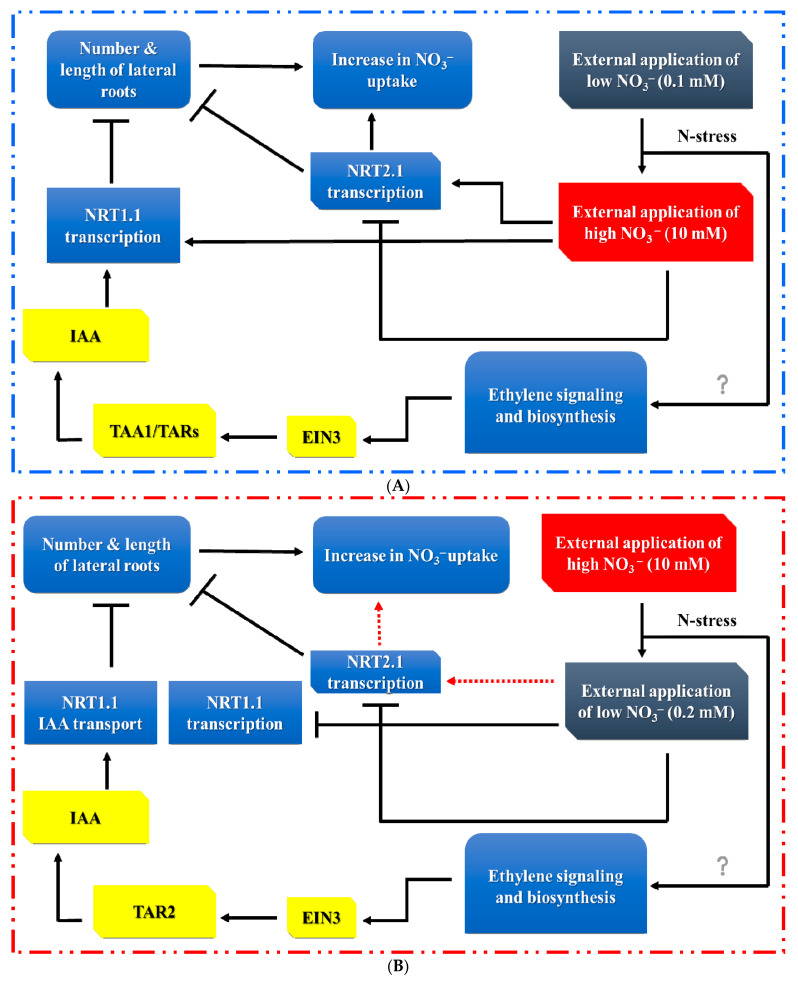
Effect of short-term ethylene biosynthesis, expression of *NRT* genes, and *Arabidopsis* root system architecture. (**A**) Changes in ethylene via nitrate concentration upregulate *AtNRT1.1* and down-regulate *AtNRT2.1* (adapted from [149]). (**B**) Sudden change in ethylene by the deprivation of external NO_3_^−^ in the medium down-regulates the transcription of *AtNRT2.1*. According to this model, the ethylene signaling component will be involved in the de-induction of the *AtNRT2* gene (adapted from [41]). Arrows and blunted lines indicate positive and negative gene expression control, respectively. Dotted arrows show temporary regulation, while a question mark indicates an unknown signaling mechanism (?).

Several researchers have explored the short-term (≤24 h) ethylene response to changes in external nitrate availability through the expression of nitrate transporter (*NRT*) genes [146,147]. Seedlings were grown in a low-nitrate (0.1 mM) concentration solution for 5 days before being transferred to a high external concentration (10 mM) solution for 6–24 h and analyzed for NO_3_^−^/ethylene interactions [145]. Another study examined this interplay by growing seedlings in high-nitrate environments (10 mM) for 1 week before transferring them to environments with low external nitrate levels (0.2 mM) for 24 h [148]. Following a burst of ethylene production (0.5–1 h) from both stresses in the roots, the ethylene concentration gradually decreased. Moreover, the exogenous application of the ethylene synthesis precursors *AMINOCYCLOPROPANE CARBOXYLIC ACID SYNTHASE* (*ACC*) and *AMINOETHOXYVINYLGLYCINE* (*AVG*) at low and high nitrate concentrations led to the upregulation and downregulation of nitrate transporters (*AtNRT1.1* and *AtNRT2.1*) [145]. However, *etr1-3* and *ein2-1* mutants showed no sensitivity to high nitrate concentrations [145], concluding that ethylene biosynthesis and signaling are necessary for regulating both *AtNRT* genes (Figure 4A). 

A high level of nitrate may modulate the actions of ethylene on nitrate transporters and metabolism [150]. It was documented that the expression of *AtNRT2.1* rather than *AtNRT1.1* was associated with ethylene biosynthesis and signaling responses in seedlings transferred from high to low nitrate concentrations [148]. In particular, a study of the expression of *AtNRT2.1* in wild-type and mutant forms of the *ctr1-1*, *ein3-1*, and *eil-1* genes revealed that ethylene repressed *AtNRT2.1* and nitrate acquisition through one of the ethylene signaling cascade’s components [148]. Therefore, a feedback loop under nitrate deficiency is linked to ethylene biosynthesis and signaling and *AtNRT2.1* expression (Figure 4B). Consequently, the results show that ethylene biosynthesis and signaling play a role in the short-term responses to nitrate deficiency and excess via fine-tuning the expression of the *AtNRT2.1* and *AtNRT1.1* genes. This study does not demonstrate how ethylene can affect nitrate uptake by altering the root system’s long-term morphology (Figure 2).

An interesting study reveals that ethylene may serve as a signal for plant-to-plant communication in rice under high-density stress conditions when ethylene production is linked to genes involved in ethylene homeostasis [151]. Researchers found that the amount of nitrogen (N) in the soil can affect the growth of ethylene-dependent rice plants. They also found that the development of the plants was hampered in high-density situations when N was either limited (3 mM NO_3_^−^) or sufficient (10 mM NO_3_^−^).

In *Brassica juncea* plants, N availability impacts photosynthesis, stomatal conductance, and growth and influences ethylene’s evolution [152,153]. According to a field study, *ethephon* (*an ethylene-releasing chemical*) improved ethylene production and photosynthesis of plants grown with N levels of 40 and 80 mg kg^−1^ [152]. Table 4 lists all of the genes and their functions that are involved with ETH-regulated N uptake and transport.

## 6. Brassinosteroid (BR), Strigolactones (SLs), Jasmonic Acid (JA), and Salicylic Acid (SA) Modulate RSA in Response to Nitrogen Stress

An increasing body of research shows that phosphate (Pi) or boron (B) deficiency suppresses brassinosteroid (BR) levels, whereas N deprivation elevates them [158,159]. In *Arabidopsis*, nitrogen deficiency promotes BR biosynthesis by upregulating *DWF1*, *CPD*, *DWF4*, and *BR6ox2* [160,161]. Moreover, roots deficient in nitrogen elevate the expression of *CPD*, *DWF4*, and *DWF1* in maize and rapeseed, respectively [161,162,163], implying that plant species respond similarly to N deficiency by inducing BR biosynthesis.

Many plant species produce strigolactones as plant hormones [164]. Despite being primarily produced by roots, strigolactones are also synthesized in other parts of plants [165]. Furthermore, it has been reported that strigolactones’ biosynthesis is impacted by N, Pi, and S deficiencies [166,167]. SL biosynthesis can increase with the depletion of these nutrients, but distinct catalytic steps have been observed. The expression levels of *MAX3* and *MAX4* are consistently increased by N and Pi deficiency in *A. thaliana*, similar to *Oryza sativa D17* and *D10* [166,168].

Low levels of N have been demonstrated to trigger an undisclosed mechanism that reduces the concentrations of *jasmonic acid (JA) and jasmonic acid–isoleucine (JA-Ile)* in *A. thaliana* roots [169,170,171].

There is currently limited understanding of how the availability of nutrients affects the production of salicylic acid (SA) in the roots. Two recent studies have demonstrated that roots that lack both N and Pi accumulate more SA [172,173]. Despite this, little is known about the underlying mechanism. The gene functions associated with N uptake and transport regulated by BR, SA, and SL are summarized in Table 5.

## 7. Conclusions and Prospects

This review comprehensively explains the molecular mechanisms of phytohormones in nitrogen signaling. Phytohormones (*CK*, *ABA*, *AUX*, *ETH*, *BR*, *SL*, *JA*, and *SA*) are the key players that regulate several signaling pathways in response to nitrogen stress and modulate root system architecture (Figure 2). Nitrogen and phytohormones signify a considerable research gap for each defined effect on root growth and development. Researchers have reported an interaction between auxin and other phytohormones (such as *CK*, *AUX*, *ABA*, etc.) that regulates RSA [182,183,184,185]. However, it is uncertain if interactions between multiple phytohormones have a role in response to N availability.

Furthermore, it is worth exploring how phytohormone signals are influenced by N availability through crosstalk between key regulatory elements. It is a fascinating hypothesis, but many questions remain unanswered. What is the process for assessing and translating nutritional status into phytohormone signals? The process of sensing and translating takes place in which cell, tissue, or organ? Which nutrient signals are the most important? What is the effective way of transmitting phytohormones and nutrients to plant target sites? Where and how are the phytohormonal signal and nutrient-specific signals interpreted and incorporated to deliver a proper nutrient response? In conjunction with system biology approaches, genomic research possesses a great perspective to identify the most critical players in interconnected regulatory networks.

It is most advantageous to apply advanced biotechnology approaches, including genome-wide association studies, omics, and bioinformatics, to decipher the genetic response of RSA to N signals and pinpoint valuable natural alleles. In addition, the identified favorable alleles can be utilized to improve N utilization efficiency using CRISPR/Cas9 technology in crops by determining root tissue/cell-specific expression and allele-specific modification. A clear understanding of genetic regulation of nitrogen use efficiency (NUE) and breeding crops with high yields using low nitrogen fertilizer inputs will be essential in the future with the use of advanced biotechnologies and accumulated basic research findings.

## Figures and Tables

**Table 1:** List of genes and their roles associated with N stress and CK in Arabidopsis and rice.

Arabidopsis			
Gene Name	Gene ID	Functions	References
*AtGRXS1*	*At1g03020*	NO_3_^−^ upregulated *GRXs* in shoots to modulate primary root (PR) elongation through CK signaling	[75,76]
*AtGRXS3*	*At4g15700*		
*AtGRXS4*	*At4g15680*		
*AtGRXS5*	*At4g15690*		
*AtGRXS6*	*At3g62930*		
*AtGRXS8*	*At4g15660*		
*AtGRXS11*	*At3g62950*		
*AtIPT3*	*At4g17870*	Involved in CK biosynthesis, modulating lateral root (LR) elongation in response to partial NO_3_^−^ deficiency	[77]
*AtIPT5*	*At3g50500*		
*AtIPT7*	*At5g66880*		
*AtABCG14*	*At1g31770*	Involved in CK transport in response to N	[78]
*CYP735A2*	*At1g67110*	Involved in CK biosynthesis and modulating RSA in response to N-stress	[79]
**Rice**			
*OsIPT4*	*Os03g0810100*	Essential for CK biosynthesis dependent on the glumine-related signal	[80]
*OsIPT5*	*Os07g0211700*		
*OsIPT7*	*Os05g0551700*		
*OsIPT8*	*Os01g0688300*		

**Table 2:** List of genes and their roles associated with N stress and ABA in Arabidopsis and wheat.

Arabidopsis			
Gene Name	Gene ID	Functions	References
*AtPYL2*	*At2g26040*	Involved in ABA signaling and PR and LR growth in response to NO_3_^−^	[87]
*AtPYL4*	*At2g38310*		
*AtABI1*	*At4g26080*		
*AtPYL1*	*At5g46790*		
*AtABI2*	*At5g57050*		
*AtHAB1*	*At1g72770*		
*AtPP2Ca*	*At3g11410*		
*AtPYR1*	*At4g17870*		
*AtSnRK2.2*	*At3g50500*	Involved in ABA signaling and PR growth in response to NO_3_^−^	[89,100]
*AtSnRK2.3*	*At5g66880*		
*AtSnRK2.6*	*At4g33950*		
**Wheat**			
*TaGS2-2Ab*	*TraesCS2A02g500400*	Nitrogen use efficiency and ABA signaling	[49]
*TaNAR2.1*	*TraesCS6D02G193100*	Nitrogen uptake and assimilation, ABA biosynthesis	[101,102]
*TaNAR2.2*	*TraesCS5D02G506100*		
*TaNRT2.1*	*TraesCS6A02G030900*		
*TaNRT2.2*	*TraesCS6D02G035800*		
*TaWabi5*	*TraesCS5B02G235600*	Involved in ABA signaling in response to low N	[103]
*TaBG1*	*TraesCS6A02G048200*	Involved in ABA biosynthesis in response to low N	

**Table 3:** List of genes and their roles associated with N stress and AUX in Arabidopsis, maize, rice, and wheat.

Arabidopsis			
Gene Name	Gene ID	Functions	References
*AtGOXL3*	*At1g75620*	N-regulated putative auxin efflux carries	[136]
*AtPILS2*	*At1g71090*		
*AtPILS6*	*At5g01990*		
*AtPLT1*	*At3g20840*	Regulating cell elongation in the process of severe NO_3_^−^ deficiency and inhibiting PR elongation	[110]
*AtPLT2*	*At5g51190*		
*AtWOX5*	*At3g11260*		
*AtWRKY46*	*At2g46400*	Regulated by high NH_4_^+^ and activates auxin conjugating genes *GH3.1*, *3.6* to modulate PR elongation	[137]
*miR167a*	*At3g22886*	Regulated by glutamine and glutamate to modulate LR development	[62]
*AtARF8*	*At5g37020*		
*miR393*	*At2g39885*	NO_3_^−^-responsive *miR393/AFB3* regulatory module controlling RSA	[110]
*AtAFB3*	*At1g12820*		
*AtOBP4*	*At5g60850*	Modulating LR initiation by auxin signaling in response to NO_3_^−^ supply	[138]
*AtNAC4*	*At5g07680*		
*AtSNX1*	*At5g57090*	N-regulated auxin-transport-related gene	[35]
*AtAXR4*	*At1g54990*	Mediating LR elongation in response to local NO_3_^−^ supply	[110]
*AtPIN1*	*At1g73590*	Modulating PR elongation through mediating auxin flow in response to NO_3_^−^ supply	[132]
*AtPIN2*	*At5g06140*		
*AtPIN4*	*At2g01420*		
*AtPIN7*	*At5g01990*		
*AtAUX1*	*At2g38120*		
*AtLAX2*	*At2g21050*		
*AtLAX3*	*At1g77690*		
*AtAGL21*	*At4g37940*	Involved in auxin homeostasis and modulating LR elongation under NO_3_^−^ deficiency	[139]
*AtGH3.1*	*At2g14960*	Regulating auxin levels and NH_4_^+^ sensitivity in roots	[88]
*AtGH3.2*	*At4g37390*		
*AtGH3.3*	*At2g23170*		
*AtGH3.4*	*At1g59500*		
*AtGH3.6*	*At5g54510*		
*AtYUC3*	*At1g04610*	Promoting auxin levels in LR tips and modulating LR elongation in response to N deficiency	[140]
*AtYUC5*	*At5g43890*		
*AtYUC7*	*At2g33230*		
*AtYUC8*	*At4g28720*		
*AtTAR1*	*At1g23320*	Involved in auxin biosynthesis and LR growth in response to low N	[49]
*AtTAR2*	*At4g24670*		
*AtTAA1*	*At1g70560*		
**Maize**			
*ZmPIN1*	*Zm00001d044812*	Upregulated by local, high NO_3_^−^ in roots	[141]
*ZmPIN9*	*Zm00001d043179*		
**Rice**			
*OsDNR1*	*Os01g0178000*	Regulating auxin homeostasis, N uptake, and assimilation	[142]
*OsARF1*	*Os01g0236300*	Auxin response factors, positiveregulates N uptake	[129]
*OsARF5*	*Os02g0141100*		
*OsARF6*	*Os02g0164900*		
*OsARF17*	*Os06g0677800*		
*OsARF19*	*Os06g0702600*		
*OsARF24*	*Os12g0479400*		
*OsARF25*	*Os12g0613700*		
*OsPIN1*	*Os11g0137000*	Auxin efflux carriers, regulated by local NO_3_^−^ supply and modulate LR growth and seminal root (SR) elongation	[107]
*OsPIN2*	*Os06g0660200*		
*OsPIN5*	*Os01g0919800*		
*OsPIN9*	*Os01g0802700*		
*OsPIN10*	*Os05g0576900*		
*OsAUX1*	*Os01g0856500*	Auxin influx carriers, regulated by local NO_3_^−^ supply and modulate LRP establishment	[127]
*OsAXR4*	*Os11g0544100*		
**Wheat**			
*TaTAR2.1-3A*	*TraesCS3A02G093000*	Involved in auxin biosynthesis in response to low N	[49]
*TaTAR2.5*	*TraesCS1B02G133900*		

**Table 4:** List of genes and their roles associated with N stress and ETH in Arabidopsis.

Arabidopsis			
Gene Name	Gene ID	Functions	References
*AtCTR1*	*At5g03730*	Involved in ETH signaling in response to low NO_3_^−^	[154]
*AtEIL1*	*At2g27050*		
*AtEIN3*	*At3g20770*		
*AtEIN2*	*At5g03280*	Involved in NO_3_^−^-dependent root growth and branching	[155]
*AtETR1*	*At1g66340*		
*AtACS2*	*At1g01480*	Involved in ETH biosynthesis in response to high NO_3_^−^	[156,157]
*AtACS4*	*At2g22810*		
*AtACS5*	*At5g65800*		
*AtACS6*	*At4g11280*		
*AtACS7*	*At4g26200*		
*AtACS8*	*At4g37770*		
*AtACS11*	*At4g08040*		
*AtACO1*	*At2g19590*		
*AtACO2*	*At1g62380*		

**Table 5:** List of genes and their roles associated with N stress and BR, SA, and SL in Arabidopsis, maize, and rice.

Arabidopsis				
Hormones	Gene Name	Gene ID	Functions	References
BR	*AtBAK1*	*At4g33430*	Involved in BR signaling and low NO_3_^−^ promoting PR elongation	[158,174]
	*AtBSK3*	*At4g00710*		
	*AtBR6ox2*	*At3g30180*	N deficiency regulating BR biosynthesis genes	[175]
	*AtDWF4*	*At3g50660*		
	*AtCPD*	*At5g05690*		
	*AtDWF1*	*At3g19820*	Involved in BR biosynthesis and root foraging response	[161]
SA	*AtSIZ1*	*At5g60410*	Regulating SA levels and activating *NIA1* and *NIA2*	[176]
**Maize**				
BR	*ZmBRI1*	*Zm00001d011721*	Involved in BR signaling and PR elongation in response to low NO_3_^−^ stress	[177]
	*ZmDET2*	*Zm00001d007910*	N-regulated BR-biosynthesis-related genes	[178]
	*ZmBZR1*	*Zm00001d046305*		
	*ZmCPD*	*Zm00001d052475*		
	*ZmDWF4*	*Zm00001d028325*		
**Rice**				
BR	*OsRAVL1*	*Os04g0581400*	Involved in BR signaling and involved in BR signaling and regulating *OsAMT1;2* and NH_4_^+^ uptake	[179]
	*OsBZR1*	*Os07g0580500*		
	*OsMADS23*	*Os08g0431900*	BR biosynthesis gene *OsBRD1* involved in NH_4_^+^-inhibited SR elongation	[180]
	*OsMADS25*	*Os04g0304400*		
	*OsMADS27*	*Os02g0579600*		
	*OsMADS57*	*Os02g0731200*		
	*OsMADS61*	*Os04g0461300*		
	*miR444*	*Os02g0731300*		
	*OsBRD1*	*Os03g0602300*		
SL	*OsSPL14*	*Os08g0509600*	SL signaling target proteins and modulating SR elongation	[80]
	*OsSPL17*	*Os09g0491532*		
	*OsD10*	*Os01g0746400*	Involved in SL biosynthesis in response to NO_3_^−^	[181]
	*OsD17*	*Os04g0550600*		

## Data Availability

Not applicable.
